# Flow and heat transport phenomenon for dynamics of Jeffrey nanofluid past stretchable sheet subject to Lorentz force and dissipation effects

**DOI:** 10.1038/s41598-021-02212-3

**Published:** 2021-11-25

**Authors:** Faisal Shahzad, Dumitru Baleanu, Wasim Jamshed, Kottakkaran Sooppy Nisar, Mohamed R. Eid, Rabia Safdar, Khadiga Ahmed Ismail

**Affiliations:** 1 Department of Mathematics, Capital University of Science and Technology (CUST), Islamabad, 44000 Pakistan; 2 Institute of Space Sciences, 077125 Magurele-Bucharest, Romania; 3 Department of Medical Research, China Medical University Hospital, China Medical University, Taichung, 40447 Taiwan; 4 Department of Mathematics, Cankaya University, 06530 Ankara, Turkey; 5 Department of Mathematics, College of Arts and Sciences, Prince Sattam Bin Abdulaziz University, Wadi Aldawaser, 11991 Saudi Arabia; 6 Department of Mathematics, Faculty of Science, New Valley University, Al-Kharga, Al-Wadi Al-Gadid, 72511 Egypt; 7 Department of Mathematics, Faculty of Science, Northern Border University, Arar, 1321 Saudi Arabia; 8 Department of Mathematics, Lahore College for Women University, Lahore, 54000 Pakistan; 9 Department of Clinical Laboratory Sciences, College of Applied Medical Sciences, Taif University, P.O. Box 11099, Taif, 21944 Saudi Arabia

**Keywords:** Mathematics and computing, Physics

## Abstract

Survey of literature unveils that nanofluids are more efficient for heat transport in comparison to the traditional fluids. However, the enlightenment of developed techniques for the augmentation of heat transport in nanomaterials has considerable gaps and, consequently, an extensive investigation for aforementioned models is vital. The ongoing investigation aims to study the 2-D, incompressible Jeffrey nanofluid heat transference flow due to a stretchable surface. Furthermore, the effect of dispersion of graphene nanoparticles in base liquid ethylene glycol (EG) on the performance of flow and heat transport using the Tawari-Das model in the existence of Ohmic heating (electroconductive heating) and viscous heat dissipation is contemplated. The boundary-layer PDEs are reconstituted as ODEs employing appropriate similarity transformation. Keller-Box Method (KBM) is utilized to determine the numerical findings of the problem. Graphene conducts heat greater in rate than all of the other materials and it is a good conductor of electrical energy. Graphene/EG nanofluid is employed to look out the parametric aspects of heat transport flow, drag coefficient, and heat transference rate phenomena with the aid of graphs and tables. The numerical outcomes indicate that concentration and magnetic field abate the shear stresses for the nanofluid. An increase of Graphene nanoparticle volume fraction parameter can boost the heat transport rate. The effect of Prandtl Number is to slow down the rate of heat transport as well as decelerate the temperature. Additionally, the rate of heat transportation augments on a surface under Deborah's number. Results indicate that the temperature of the graphene-EG nanofluid is greater than the convectional fluid hence graphene-EG nanofluid gets more important in the cooling process, biosensors and drug delivery than conventional fluids.

## Introduction

On account of the limited capabilities of typical heat transport base liquids (engine oil, water, polymer solutions, and tri-ethylene-glycols) are not adequate to address today’s requirements. Consequently, the advanced form of potentially high heat transport fluids ascribed to NFs is presented and launched in the industrial sectors. NFs are an amalgamation of nanomolecules (< 100 nm) in conventional liquids that manifest higher heat transport efficiency than common liquids^1^. NFs have acquired a great deal of importance in the last few years in varied disciplines such as automobile, electronics cooling, catalysis, smart computers, solar energy, transport and biomedical, etc.^2^. Dogonchi and Ganji^3^ have examined the numerical evaluation of heat transport nanofluid flow beneath the influence of Cattaneo/Christov thermal fluxing model past a stretchable surface. Eid and Mahny^4^ concentrated on theoretical aspects of laminar flow and transport of heat of Sisko nanofluid past an exponentially stretching surface embedded in a penetrable material and tackle the problem utilizing the numerical Runge–Kutta Fehlberg technique. Shawky et al.^5^ considered the porous medium influence on magnetohydrodynamic laminar flowing with the transport of heat of Williamson nanofluid through an extending surface. Lin et al.^6^ viewed the impact of the Lorentz force and thermal radiative flow on the magnetite-water nanofluidic and heat transport via an extending rotate disk. Kumar^7^ considered the MHD heat transference convective nanofluid flow through an impetuously initiated vertical surface under the impression of thermal radiative impact. Amjad et al.^8^ presented the effect of the stagnation region in the boundary layer flow of Casson micropolar nanoliquid past a bent expanding sheet.

Study of the transport of energy utilizing the power-law liquid under the influence of magnetohydrodynamic is a significant topic in the fluid dynamic result in the wide-ranging of implementations in the diverse fields such as oil recovery, plasmas, synthetic lubricants, paints, liquid metals, alloys, oil reservoir engineering, and cosmetics. Various nonlinear constitutive connections have been inspected for the stress and the shear rate for power-law liquids. The impacts of suction/injection on pseudoplastic nanoliquid flow towards a penetrable sheet were scrutinized by Maleki et al.^9^. Mabood et al.^10^ have inspected the influence of Robinson’s constraint and Arrhenius exponential parameter law on non-Newtonian nanofluid past a thin needle with double stratification. Reddy and Lakshminarayana^11^ observed the influences of the heat radiative flowing and cross-diffusion on the laminar motion of three-dimensional motion of MHD non-Newtonian nanofluid along with an extending sheet with heat source effect. Jeffrey fluid is one of a non-Newtonian viscoelastic fluid model that portrays the most important characteristics of retardation and relaxation times. Rasool et al.^12^ address the features of magnetohydrodynamic Jeffrey nanofluid flow along a stretching surface with Darcy-Forchheimer relation. Ahmad et al.^13^ investigated via a homotopic technique the chemically reactive impact on a boundary-layer flowing of Jeffrey nanofluid through an accelerated surface.

The boundary layer laminar motion of fluid along the continuous moving surfaces with various flow characteristics has turned out to be one of the important areas of interest by diverse applications in multifarious engineering and industrial phenomenons. Especially, in plastic manufacturing, extruded polymer sheets, fiber spinning, crystal growing, emulsion coated sheets, material-handling conveyors, food production, cooling of an infinite metallic plate, and so forth. Shahzad et al.^14^ inspected the influence of suction/injection in axisymmetric heat transference flow past an exponential stretched sheet with the magnetic field features. Ibrahim and Gadisa^15^ discussed the two-dimensional flowing result in a nonlinearly moving plate by considering an Oldroyd-B fluid with heat source (sink) influences. Megahed^16^ initiated the research on a steady flowing of Maxwell fluid via an extending plate with heat source and variable viscosity using the shooting method. The influence of the magneto dipole on the flowing of ferromagnetic nanoliquid towards a flat elastic surface was scrutinized by Gowda et al.^17^.

In modern metal-working processes and metallurgical, the study of the magneto-fluid dynamics (MHD) flowing of electrical conductive is of great significance. In controlling laminar flow and energy transport of diverse liquids via a stretching sheet, MHD plays a significant role. Moreover, it has various applications in MHD generators, biomedicine, furnace structure, optical modulators, cancer tumor treatment, magnetic optical wave-length purification schemes, nanofluid MHD pumping, photosensitive controller keys, drugs transporter, magnetic drug targeting, and so forth. Ghasemi and Hatami^18^ inspected the impact of solar radiative on MHD stagnating point flowing along with an elongating surface. Patil et al.^19^ interrogated the impact of thermal radiation on unsteady MHD flowing of a Powell-Eyring nanoliquid via an extending plate. The impact of viscidness heat dissipative variations the thermal distributions by having a role as a heat generation, which has an impact on the rate of heat transit. Shateyi and Marewo^20^ determined the heat transference characteristics in the mixed convective flowing of a micro-polar liquid through an unsteady stretchable plate with viscidness dissipative flowing. Swain et al.^21^ elucidated the flow of Newtonian fluid enclosed in a penetrable material above an elongated sheet under the impact of viscous dissipation. Like viscous heat dissipation, Joule heating or Ohmic heating plays the role of heat source in viscous fluids. Aly and Pop^22^ deliberated the viscous dissipation and partial slippery aspects in the 2D flowing of hybrid nanoliquid above an accelerating sheet.

Based on the above-mentioned articles and as far as we know, MHD Jeffrey nanofluid heat transport flow above a linearly stretchable surface with Ohmic heating, viscous heat dissipation, and graphene nanoparticles suspension had not been examined. The tested nanofluid is comprised of graphene nanoparticles and ethylene glycol as the base liquid. A substantial research is being accomplished about the numerical solution of the nanofluid flow model, though very few investigators attempted to tackle the nanofluid flow problem with novel numerical method. Numeric solutions for the dimensionless stream function and dimensionless temperature is determined under the aegis of robust Keller box method. Diagrams and tables are constructed to explore the results of appropriate factors on flowing, energy, heat transport rate, and surface drag force with the aid of MATLAB program.

## Physical model

**Figure 1:**
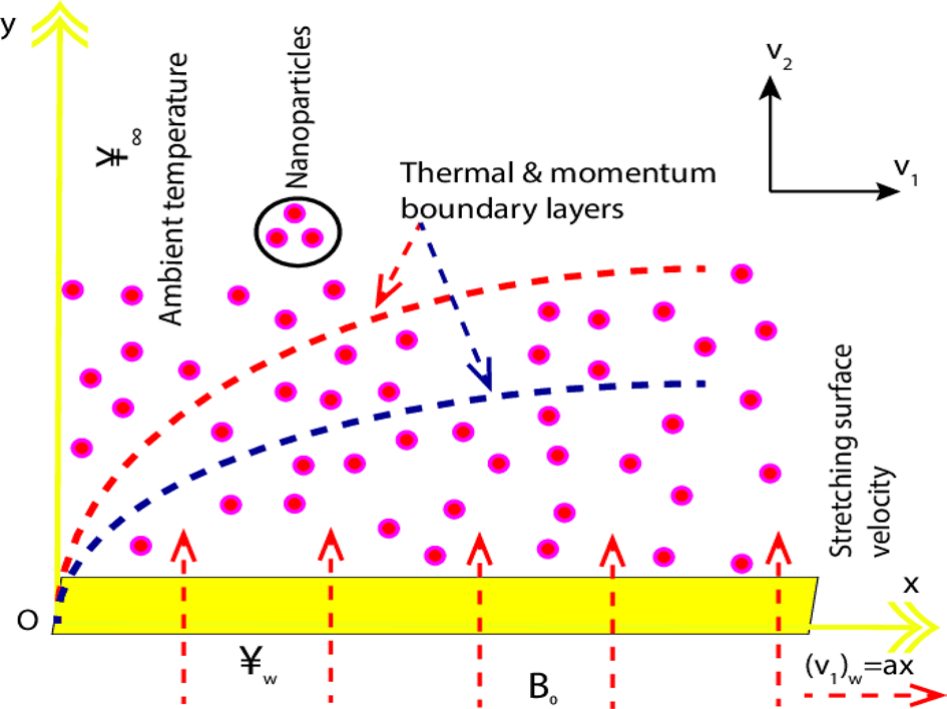
Physical flow configuration.

Two-dimensional time-independent, laminar, and incompressible electrically conducting Jeffrey nanoliquid flowing via a linear stretchable sheet with heat generation (absorption), Ohmic heating, and viscous dissipation effects are examined. Graphene is utilized as the nanomaterials while ethylene glycol is the conventional base liquid. $x -$axis is drawn alongside the horizontal stretched plate and $y -$axis is considered perpendicular to the stretchable plate. The sheet with a fixed rate $a$ and speed $(v_{1} )_{w} = ax$ is extending in the $x -$axis orientation such that nanofluid is confined in $y > 0$. Transverse magnetism field with strength $B_{0}$ is utilized in $y -$direction as exhibited in Fig. 1. An induced magnetism field is not alleged because of the contemplation of an insignificant small Reynolds number. The sheet is observed to attain a temperature ${{\yen}}_{ w}$ in the square form at sheet $y = 0$ i.e., ${{\yen}}_{w } = A(x/L)^{2} + {{\yen}}_{\infty }$.

The model submitted is governed by the continuity, momentum, and energy equations of the boundary-layer below

1 $$\frac{{\partial v_{1} }}{\partial x} + \frac{{\partial v_{2} }}{\partial y} = 0,$$

2 $$\left( {v_{1} \frac{{\partial v_{1} }}{\partial x} + v_{2} \frac{{\partial v_{1} }}{\partial y}} \right) = \frac{{ \nu_{nf } }}{{\left( {1 + \lambda_{1} } \right)}}\left[ {\frac{{\partial^{2} v_{1} }}{{\partial y^{2} }} + \lambda_{2} \left( {\begin{array}{*{20}c} {v_{1} \frac{{v_{2} \partial^{3 } v_{1} }}{{\partial x\partial^{2 } y}} - \frac{{\partial v_{1} }}{\partial x}\frac{{\partial^{2 } v_{1} }}{{\partial y^{2 } }}} \\ { + \frac{{\partial v_{1} }}{\partial y}\frac{{\partial^{2 } v_{1} }}{\partial x\partial y} + v_{2} \frac{{\partial^{3} v_{1} }}{{\partial y^{3} }}} \\ \end{array} } \right)} \right] - \frac{{\sigma_{nf} }}{{\rho_{nf} }}B_{0}^{2} v_{1} ,$$

3 $$v_{1} \frac{{\partial {{\yen}}}}{\partial x} + v_{2} \frac{{\partial {{\yen}}}}{\partial y} = \frac{{ k_{nf} }}{{(\rho c_{p} )_{nf} }} \frac{{\partial^{2} {{\yen}}}}{{\partial y^{2} }} + \frac{{ \mu_{nf} }}{{(\rho c_{p} )_{nf} }}(\frac{{\partial v_{1} }}{\partial y})^{2} + \frac{{\sigma_{nf} }}{{(\rho c_{p} )_{nf} }}B_{0}^{2} v_{1}^{2} - \frac{Q}{{(\rho c_{p} )_{nf} }}\left( {{{\yen}} - {{\yen}}_{\infty } } \right).$$

The limit constraints revealed by the model's physics are:

4 $$\left. {\begin{array}{*{20}c} {v_{1} = (v_{1} )_{w } ,\;v_{2} = 0,\; {{\yen}} = {{\yen}}_{w} \; {\text{at}}\; y = 0,} \\ {v_{1} \to 0,\;\frac{{\partial v_{1} }}{\partial y } \to 0, \;{{\yen}} \to {{\yen}}_{\infty } \;{\text{as}}\;y \to \infty ,} \\ \end{array} } \right\}$$

The speed comprises two constituent parts for every orientation, i.e., $v_{1}$ and $v_{2}$ in $x$ and $y$ orientations, separately. Table 1 encapsulates the material parameters for the Jeffrey nanomaterial^23,24^. Table 2 yields the thermo-physical attributes of the standard fluid along with nanomolecules^25^.

**Table 1:** Thermophysical attributes for Jeffrey nanofluid.

Properties	Nanofluid
Dynamic viscosity	$\mu_{nf} = \mu_{f} \left( {1 - \phi } \right)^{ - 2.5}$
Density	$\rho_{nf} = \left( {1 - \phi } \right)\rho_{f} + \phi \rho_{s}$
Heat capacity	$(\rho C_{p} )_{nf} = \left( {1 - \phi } \right)\left( {\rho C_{p} } \right)_{f} + \phi \left( {\rho C_{p} } \right)_{s}$
Thermal conductivity	$\frac{{\kappa_{nf} }}{{\kappa_{f} }} = \frac{{\left[ {\left( {\kappa_{s} + (m - 1)\kappa_{f} } \right) - (m - 1)\phi (\kappa_{f} - \kappa_{s} )} \right]}}{{\left[ {(\kappa_{s} + (m - 1)\kappa_{f} ) + \phi (m - 1)(\kappa_{f} - \kappa_{s} )} \right]}}$
Electrical condectivity	$\frac{{\sigma_{nf} }}{{\sigma_{f} }} = 1 + \frac{{3(\sigma_{s} - \sigma_{f} )\phi }}{{\left( {\sigma_{s} + 2\sigma_{f} } \right) - \left( {\sigma_{s} + \sigma_{f} } \right)\phi }}$

**Table 2:** Thermo-physical attributes of ethylene glycol and graphene.

Physical properties	ρ (kg/m^3^)	C_p_ (J/kgK)	Κ (W/mK)	σ (S.m)^−1^
Graphene	2250	2100	2500	1 × 10^−7^
Ethylene glycol	1114	2415	0.252	5.5 × 10^−6^

The stream function $\Psi \left( {x,y} \right) = \Psi$ is such that $v_{1} = \frac{\partial \Psi }{{\partial y}}$ and $v_{2} = - \frac{\partial \Psi }{{\partial x}}$. By announcing the subsequent dimensionless quantities

5 $${\Upsilon } = \sqrt {\frac{a}{\nu }} y, \Psi = - \sqrt {a\nu } x F\left( {\Upsilon } \right), \Theta \left( {\Upsilon } \right) = \frac{{{{\yen}} - {{\yen}}_{\infty } }}{{{{\yen}}_{w} - {{\yen}}_{\infty } }},\;v_{1} = axF^{\prime } \left( {\Upsilon } \right),\; v_{2} = - \sqrt {a\nu } F\left( {\Upsilon } \right),$$

then the Eqs. (1)$-$(3) reduce to:

6 $$F^{\prime \prime \prime } - \frac{{\varepsilon_{2} }}{{\varepsilon_{1} }}\left( {1 + \lambda_{1} } \right)\left[ {(F^{{{\prime } }} )^{2} - FF^{\prime \prime } } \right] + \beta \left[ {( F^{\prime \prime } )^{2} - FF^{iv } } \right] - \left( {1 + \lambda_{1 } } \right)\frac{{\varepsilon_{5} }}{{\varepsilon_{2} }}MF^{{\prime }} = 0,$$

7 $$\Theta^{{{\prime \prime }}} + \frac{{\varepsilon_{3} }}{{\varepsilon_{4} }}Pr\left( {F\Theta^{{\prime }} - 2\Theta F^{{\prime }} } \right) + \frac{{\varepsilon_{1} }}{{\varepsilon_{4} }}PrEcF^{{{\prime \prime }}} + \frac{1}{{\varepsilon_{4} }}EcPrM(F^{{\prime }} )^{2} + \varepsilon_{3} Pr\gamma^{*} \Theta = 0.$$

The affined boundary constraints are:

8 $$\left. {\begin{array}{*{20}c} {F\left( 0 \right) = 0, \;F^{{\prime }} \left( 0 \right) = 1,\; {\Theta }\left( 0 \right) = 1 \quad {\text{at}}\quad {\Upsilon } = 0,} \\ {F^{{\prime }} \left( {\Upsilon } \right) \to 0,\;F^{{{\prime \prime }}} \left( {\Upsilon } \right) \to 0, \;{\Theta }\left( {\Upsilon } \right) \to 0 \quad {\text{as}}\quad {\Upsilon } \to \infty .} \\ \end{array} } \right\}$$

Dimensionless variables associated with the above equations are expressed as

9 $$\left. {\begin{array}{*{20}c} {Pr = \frac{{\mu_{f} (C_{p} )_{f} }}{{k_{f} }}\;\left( {{\text{Prandtlnumber}}} \right),} \\ {M = \frac{{\sigma_{f} B_{0}^{2} }}{{a\rho_{f} }} \;\left( {{\text{magneticfieldparameter}}} \right),} \\ {\beta = a\lambda_{2} \;\left( {{\text{Deborahnumber}}} \right),} \\ {\gamma^{*} = \frac{Q}{{a(\rho C_{p} )_{nf} }} \;\left( {{\text{heatsource}}/{\text{sinkparameter}}} \right),} \\ {Ec = \frac{{l^{2} a^{2} }}{{A(C_{p} )_{f} }} \;\left( {{\text{Eckertnumber}}} \right),} \\ {\varepsilon_{1} = (1 - \phi_{Graphene} )^{ - 2.5} ,} \\ {\varepsilon_{2} = \left[ {\left( {1 - \phi_{Graphene} } \right) + \phi_{Graphene} \frac{{\rho_{EG} }}{{\rho_{Graphene} }}} \right],} \\ {\varepsilon_{3} = \left[ {\left( {1 - \phi_{Graphene} } \right) + \phi_{Graphene} \frac{{(\rho C_{p} )_{EG} }}{{(\rho C_{p} )_{Graphene} }}} \right],} \\ {\varepsilon_{4} = \frac{{\left( {k_{Graphene} + \left( {m - 1} \right)k_{EG} } \right) + \left( {m - 1} \right)\phi_{Graphene} \left( {k_{Graphene} - k_{EG} } \right)}}{{\left( {k_{Graphene} + \left( {m - 1} \right)k_{EG} } \right) - \left( {m - 1} \right)\phi \left( {k_{Graphene} - k_{EG} } \right)}},} \\ {\varepsilon_{5} = 1 + \frac{{3\left( {\sigma_{Graphene} - \sigma_{EG} } \right)\phi_{Graphene} }}{{\left( {\sigma_{Graphene} + 2\sigma_{EG} } \right) - \left( {\sigma_{Graphene} - \sigma_{EG} } \right)\phi_{Graphene} }}.} \\ \end{array} } \right\}$$

The valuable quantities from the engineering applications viewpoint in the course of this study are the drag force $C_{f }$ and the heat transport rate $Nu_{x}$ that are expressed as

10 $$C_{f} = \frac{{2 \tau_{w} }}{{\rho_{f} (v_{1} )_{w }^{2 } }}, \;Nu_{x} = \frac{{xq_{ w} }}{{k_{f} \left( {{{\yen}}_{w} - {{\yen}}_{ \infty } } \right)}},$$

where $\tau_{w} = \mu_{nf} \left( {\frac{{\partial v_{1} }}{\partial y}} \right)$ is the surface shear stress and $q_{w} = - k_{nf} \left( {\frac{{\partial {{\yen}}}}{\partial y}} \right)$ is the sheet heat fluxing. Invoking the nondimensional variables announced prior, the Eq. (10) can be delineated as:

11 $$\begin{array}{*{20}l} {} \hfill & {C_{f} Re_{x}^{0.5} = (1 - \phi )^{ - 2.5} f^{\prime\prime}\left( 0 \right),} \hfill & {Re_{x}^{ - 0.5} Nu_{x} = - \frac{{k_{nf} }}{{k_{f} }}{{\Theta^{\prime}}}\left( 0 \right),} \hfill \\ \end{array}$$

herein $Re_{x} = \frac{{(v_{1} )_{w} x}}{{\nu_{f} }}$ signifies Reynolds number.

## Numerical process

**Figure 2:**
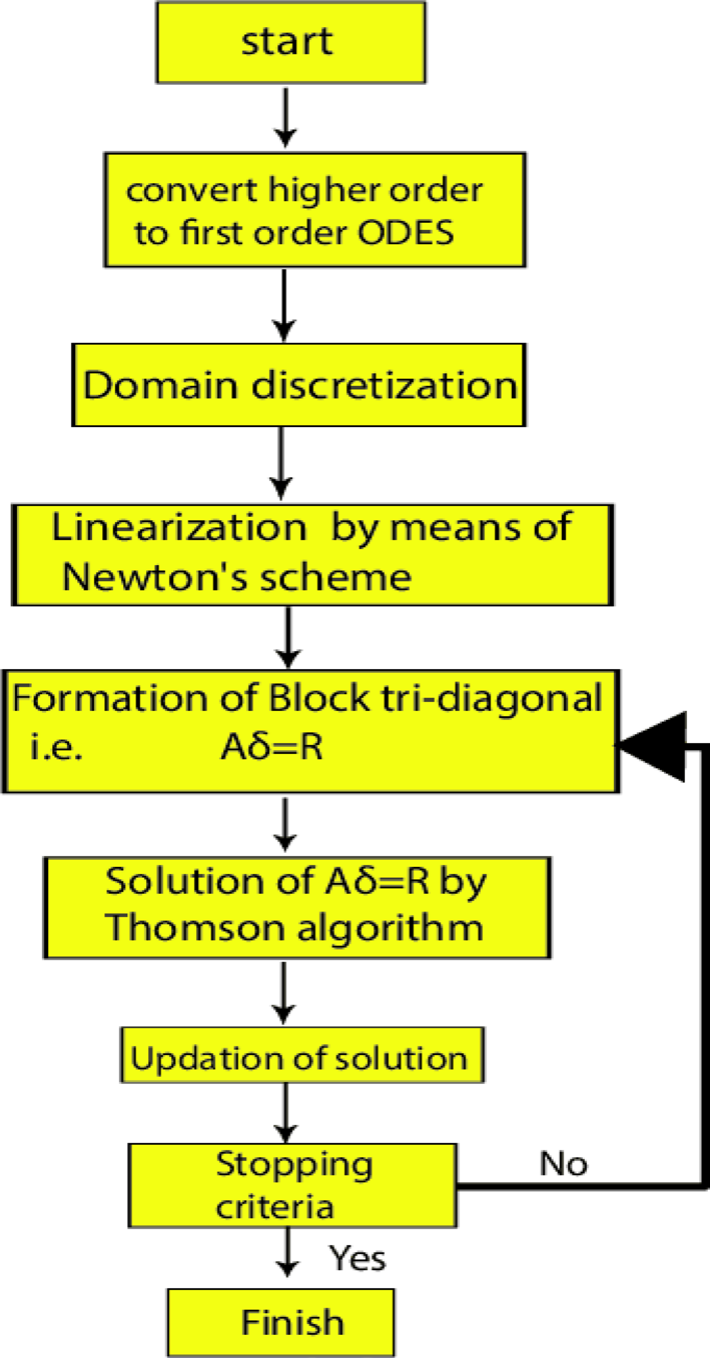
Flow chart of the current methodology.

The system of governing ODEs is nonlinear and coupled. The governing ODEs (6) and (7) with the endpoint condition (8) are tackled numerically by employing the robust Keller-box method^26,27^ utilizing MATLAB software which is dependent on the finite-difference procedure. The solution technique of the Keller box method is summed up in the flow chart in Fig. 2:

## Numerical procedure

We initiate dependent variables $\hat{v}_{1}$, $\tilde{v}_{2}$, $\tilde{v}_{3}$ and $\tilde{g}$ so that

12 $$\frac{dF}{{d{\Upsilon }}} = \hat{v}_{1} , \frac{{d\hat{v}_{1} }}{{d{\Upsilon }}} = \tilde{v}_{2} , \frac{{d\hat{v}}}{{d{\Upsilon }}} = \tilde{v}_{3} , \frac{{d{\Theta }}}{{d{\Upsilon }}} = \tilde{g}.$$

Thus Eqs. (6) and (7) might be arranged as

13 $$- \beta F\frac{{d\tilde{v}_{3} }}{{d{\Upsilon }}} + \tilde{v}_{3} - \frac{{\varepsilon_{2} }}{{\varepsilon_{1} }}\left( {1 + \lambda_{1} } \right)\left[ {\hat{v}_{1}^{2} - F\tilde{v}_{2} } \right] + \beta \tilde{v}_{2}^{2} - \frac{1}{{\varepsilon_{1} }}\left( {1 + \lambda_{1} } \right)M\hat{v}_{1} = 0$$

and

14 $$\frac{{d\tilde{v}_{g} }}{{d{\Upsilon }}} + \frac{{\varepsilon_{3} }}{{\varepsilon_{4} }}Pr\left( {F\tilde{v}_{g} - 2\hat{v}_{1} \Theta } \right) + \frac{{\varepsilon_{1} }}{{\varepsilon_{4} }}PrEc \tilde{v}_{2}^{2} + \frac{1}{{\varepsilon_{4} }}MPrEc \hat{v}_{1}^{2} + \varepsilon_{3} Pr\gamma^{*} \Theta = 0.$$

The endpoint constraints same way are altered and changes into the form

15 $$\left. {\begin{array}{*{20}c} {F \left( 0 \right) = 0, \;\hat{v}_{1} \left( 0 \right) = 1, \;{\Theta } \left( 0 \right) = 1,} \\ {\hat{v}_{1} \to 0, \;\tilde{v}_{2} \to 0, \;{\Theta } \to 0\quad {\text{as}}\quad {\Upsilon } \to \infty .} \\ \end{array} } \right\}$$

**Figure 3:**
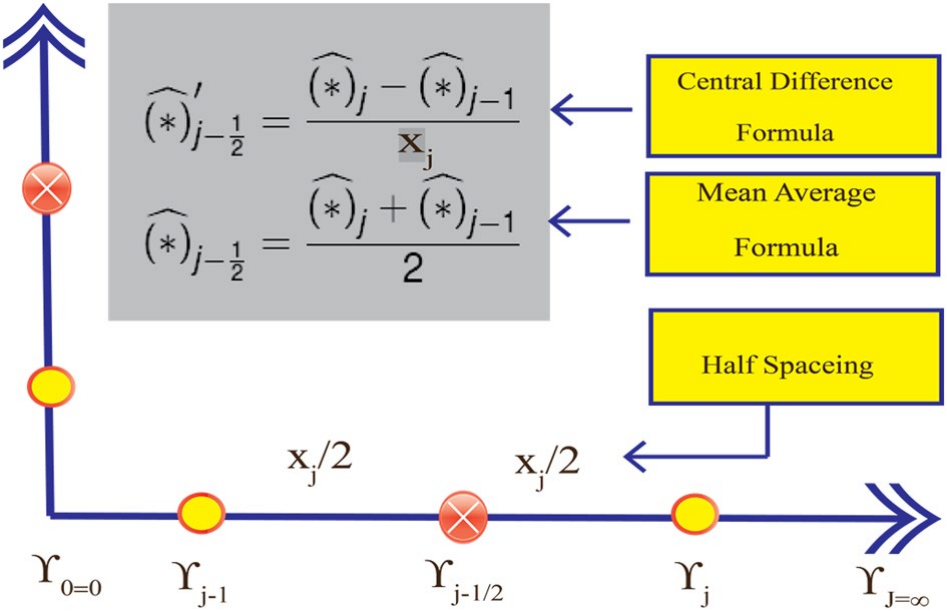
Finite difference space grid.

The net on ${\Upsilon }$ is defined employing the succeeding nodes (see Fig. 3):

$${\Upsilon }_{0} = 0,\quad {\Upsilon }_{j} = {\Upsilon }_{j - 1} + x_{j} ,x_{j} = \frac{{{\Upsilon }_{J} - {\Upsilon }_{0} }}{{{\Upsilon }_{p} }} \quad j = 1, 2,3 \ldots ,J, \quad {\Upsilon }_{J} = {\Upsilon }_{\infty } ,$$

in which $x_{j}$ symbolizes the uniform step size and ${\Upsilon }_{p}$ specifies the numbers of mesh points. The first-order system is approximated employing central difference derivates and midpoint on an arbitrary rectangular mesh. This leads to a subsequent system:

16 $$\frac{{F_{j } - F_{j - 1 } }}{{x_{j} }} = \frac{{\hat{v}_{1j} + \hat{v}_{1j - 1} }}{2},$$

17 $$\frac{{\hat{v}_{1j} - \hat{v}_{1 j - 1} }}{{x_{j } }} = \frac{{\tilde{v}_{2j} + \tilde{v}_{2 j - 1} }}{2},$$

18 $$\frac{{\tilde{v}_{2j} - \tilde{v}_{2j - 1} }}{{x_{j} }} = \frac{{\tilde{v}_{3j} + \tilde{v}_{3j - 1} }}{2},$$

19 $$\frac{{{\Theta }_{j } - {\Theta }_{j - 1 } }}{{x_{j} }} = \frac{{\tilde{g}_{j } + \tilde{g}_{j - 1 } }}{2},$$

20 $$\left. {\begin{array}{*{20}c} \frac{{\tilde{v}_{3j} + \tilde{v}_{3j - 1}}}{2} - \frac{{\varepsilon_{2} }}{{\varepsilon_{1} }}\left( {1 + \lambda_{1} } \right)\left[ {\left( {\frac{{\hat{v}_{1j} + \hat{v}_{1j - 1}}}{2}} \right)^{2} - \left( {\frac{{F_{j} + F_{j - 1} }}{2}} \right)\left( {\frac{{\tilde{v}_{2j} + \tilde{v}_{2j - 1} }}{2}} \right)} \right] \\ + \beta \left[ {\left( {\frac{{\tilde{v}_{2j} + \tilde{v}_{2j - 1}}}{2}} \right)^{2} - \left( {\frac{{F_{j} + F_{j - 1} }}{2}} \right)\left( {\frac{{\tilde{v}_{3j} - \tilde{v}_{3j - 1}}}{{x_{j} }}} \right)} \right] \\ - \frac{1}{{\varepsilon_{1} }}M\left( {1 + \lambda_{1} } \right)\left( {\frac{{\hat{v}_{1j} + \hat{v}_{1j - 1}}}{2}} \right) = 0, \\ \end{array} } \right\}$$

21 $$\left. {\begin{array}{*{20}c} {\frac{{\tilde{g}_{j} + \tilde{g}_{j - 1} }}{{x_{j} }} + \frac{{\varepsilon_{3} }}{{\varepsilon_{4} }}Pr\left( {\frac{{F_{j} + F_{j - 1} }}{2}} \right)\left( {\frac{{\tilde{g}_{j} + \tilde{g}_{j - 1} }}{2}} \right) - 2\frac{{\varepsilon_{3} }}{{\varepsilon_{4} }}Pr\left( {\frac{{\hat{v}_{1j} + \hat{v}_{1j - 1} }}{2}} \right)} \\ {\left( {\frac{{{\Theta }_{j} + {\Theta }_{j - 1} }}{2}} \right) + \frac{{A_{1 } }}{{A_{4 } }}PrEc\left( {\frac{{\tilde{v}_{2j} + \tilde{v}_{2j - 1} }}{2}} \right)^{2} + \frac{1}{{A_{4 } }}MPrEc\left( {\frac{{\hat{v}_{1j} + \hat{v}_{1j - 1} }}{2}} \right)^{2} } \\ { + \varepsilon_{3} Pr\gamma^{*} \left( {\frac{{{\Theta }_{j} + {\Theta }_{j - 1} }}{2}} \right) = 0.} \\ \end{array} } \right\}$$

Linearization mechanism of the acquired nonlinear difference Eqs. (16)–(21) is executed with the assistance of Newton’s method and the subsequent substitution is unveiled:

22 $$\left. {\begin{array}{*{20}c} {f_{j }^{n + 1} = f_{j }^{n } + \delta f_{j}^{n} ,\quad \hat{v}_{1j}^{n + 1} = \hat{v}_{1j}^{n} + \delta \hat{v}_{1j}^{n} ,\quad \tilde{v}_{2j}^{n + 1} = \tilde{v}_{2j}^{n } + \delta \tilde{v}_{2j}^{n} ,} \\ {\tilde{v}_{3j}^{n + 1} = \tilde{v}_{3j}^{n} + \delta \tilde{v}_{3j}^{n} ,\quad \tilde{g}_{j }^{n + 1} = \tilde{g}_{j}^{n} + \delta \tilde{g}_{j}^{n} ,\quad {\Theta }_{j }^{n + 1} = {\Theta }_{j}^{n} + \delta {\Theta }_{j}^{n} .} \\ \end{array} } \right\}$$

Utilize this procedure in Eqs. (16)–(21) and disregarding the truncation errors term in $\delta^{{\prime }} s$

23 $$\delta F_{j } - \delta F_{j - 1 } - \frac{{h_{j } }}{2}\left( {\delta \hat{v}_{1j} + \delta \hat{v}_{1j - 1} } \right) = (r_{1} )_{j } ,$$

24 $$\delta \hat{v}_{1j} - \delta \hat{v}_{1j - 1} - \frac{{x_{j} }}{2}\left( {\delta \hat{v} _{j} + \delta \hat{v} _{j - 1} } \right) = (r_{2} )_{j} ,$$

25 $$\delta \tilde{v}_{2j} - \delta \tilde{v}_{2j - 1} - \frac{{x_{j} }}{2}\left( {\delta \tilde{v}_{3j} + \delta \tilde{v}_{3j - 1} } \right) = (r_{3} )_{j } ,$$

26 $$\delta {\Theta }_{j } - \delta {\Theta }_{j - 1 } - \frac{{x_{j} }}{2}\left( {\delta \tilde{g}_{j } + \delta \tilde{g}_{j - 1 } } \right) = (r_{4} )_{j } ,$$

27 $$\begin{aligned} & (\zeta_{1} )_{j} \delta \tilde{v}_{3j} + (\zeta_{2} )_{j} \delta \tilde{v}_{3j - 1} + (\zeta_{3} )_{j} \delta F_{j} + (\zeta_{4} )_{j} \delta F_{j - 1} + (\zeta_{5} )_{j} \delta \tilde{v}_{2j} + (\zeta_{6} )_{j} \delta \tilde{v}_{2j - 1} \\ & \quad + (\zeta_{7} )_{j} \delta \hat{v}_{1j} + (\zeta_{8} )_{j} \delta \hat{v}_{1j - 1} = (r_{5} )_{j} , \\ \end{aligned}$$

28 $$\begin{aligned} & (\xi_{1} )_{j} \delta \tilde{g}_{j} + (\xi_{2} )_{j} \delta \tilde{g}_{j - 1} + (\xi_{3} )_{j} \delta F_{j} + (\xi_{4} )_{j} \delta F_{j - 1} + (\xi_{5} )_{j} \delta \hat{v}_{1j} + (\zeta_{6} )_{j} \delta \hat{v}_{1j - 1} + (\xi_{7} )_{j} \delta {\Theta }_{j} \\ & \quad + (\xi_{8} )_{j} \delta {\Theta }_{j - 1} + (\xi_{9} )_{j} \delta \tilde{v}_{2j} + (\xi_{10} )_{j} \delta \tilde{v}_{2j - 1} = (r_{6} )_{j} , \\ \end{aligned}$$

where

29 $$\left. \begin{array}{*{20}c} {(\zeta_{1} )_{j} = - \frac{\beta }{2}\left( {F_{j} + F_{j - 1} } \right) + \frac{{x_{j} }}{2},\quad (\zeta_{2} )_{j} = \frac{\beta }{2}\left( {F_{j} + F_{j - 1} } \right) + \frac{{x_{j} }}{2},} \\ {(\zeta_{3} )_{j} = - \frac{\beta }{2}\left( {\tilde{v}_{3j} + \tilde{v}_{3j - 1}} \right) + \frac{{\varepsilon_{2} }}{{\varepsilon_{1} }}\frac{{x_{j} }}{4}\left( {1 + \lambda_{1} } \right)\left( {\tilde{v}_{2j} + \tilde{v}_{2j - 1} } \right) = (\zeta_{4} )_{j} ,} \\ {(\zeta_{5} )_{j} = \frac{{\varepsilon_{2} }}{{\varepsilon_{1} }}\frac{{x_{j} \left( {1 + \lambda_{1} } \right)\left( {F_{j} + F_{j - 1} } \right)}}{4} + \frac{{\beta x_{j} \left( {\tilde{v}_{2j} + \tilde{v}_{2j - 1}} \right)}}{2} = (\zeta_{6} )_{j} ,} \\ {(\zeta_{7} )_{j} = - \frac{{\varepsilon_{2} }}{{\varepsilon_{1} }}\frac{{x_{j} \left( {1 + \lambda_{1} } \right)\left( {\hat{v}_{1j} + \hat{v}_{1j - 1} } \right)}}{2} - \frac{1}{{\varepsilon_{1} }}\frac{{Mx_{j} \left( {1 + \lambda_{1} } \right)}}{2} = (\zeta_{8} )_{j} ,} \\ {(r_{5} )_{j} = \frac{{\left( {\tilde{v}_{3j} + \tilde{v}_{3j - 1}} \right)}}{2}\left( {\beta \left( {F_{j} + F_{j - 1} } \right) - x_{j} } \right) - \frac{{\varepsilon_{2} }}{{\varepsilon_{1} }}\frac{{x_{j} \left( {1 + \lambda_{1} } \right)\left( {F_{j} + F_{j - 1} } \right)\left( {\tilde{v}_{2j} + \tilde{v}_{2j - 1} } \right)}}{4}} \\ { - \frac{{\beta x_{j} (\tilde{v}_{2j} + \tilde{v}_{2j - 1})^{2} }}{4} + \frac{{\varepsilon_{2} }}{{\varepsilon_{1} }}\frac{{x_{j} \left( {1 + \lambda_{1} } \right)(\hat{v}_{1j} + \hat{v}_{1j - 1} )^{2} }}{4} + \frac{1}{{\varepsilon_{1} }}\frac{{Mx_{j} \left( {1 + \lambda_{1} } \right)\left( {\hat{v}_{1j} + \hat{v}_{1j - 1}} \right)}}{2},} \\ \end{array} \right\}$$

30 $$\left. \begin{aligned} (\xi_{1} )_{j} & = 1 + \frac{{\varepsilon_{3} }}{{\varepsilon_{4} }}\frac{{Prx_{j} \left( {F_{j} + F_{j - 1} } \right)}}{4},\quad (\xi_{2} )_{j} = (\xi_{1} )_{j} - 2, \\ (\xi_{3} )_{j} & = \frac{{\varepsilon_{3} }}{{\varepsilon_{4} }}\frac{{Prx_{j} \left( {\tilde{g}_{j} + \tilde{g}_{j - 1} } \right)}}{4} = (\xi_{4} )_{j} , \quad (\xi_{5} )_{j} = - \frac{{\varepsilon_{3} }}{{\varepsilon_{4} }}\frac{{Prx_{j} \left( {{\Theta }_{j} + {\Theta }_{j - 1} } \right)}}{2} \\ & \quad + \frac{1}{{\varepsilon_{4} }}\frac{{MPrEcx_{j} \left( {\hat{v}_{1j} + \hat{v}_{1j - 1}} \right)}}{2} + \varepsilon_{3} Pr\gamma^{*} \left( {\frac{{{\Theta }_{j} + {\Theta }_{j - 1} }}{2}} \right) = (\xi_{6} )_{j} , \\ (\xi_{7} )_{j} & = - \frac{{\varepsilon_{4} }}{{A_{5} }}\frac{{Prx_{j} \left( {\hat{v}_{1j} + \hat{v}_{1j - 1}} \right)}}{2} = (\xi_{8} )_{j} , \\ (\xi_{9} )_{j} & = \frac{{\varepsilon_{1} }}{{\varepsilon_{4} }}\frac{{PrEcx_{j} \left( {\tilde{v}_{2j} + \tilde{v}_{2j - 1} } \right)}}{2} = (\xi_{10} )_{j} , \\ (r_{6} )_{j} & = - \frac{{\varepsilon_{3} }}{{\varepsilon_{4} }}\frac{{Prx_{j} \left( {F_{j} + F_{j - 1} } \right)\left( {\tilde{g}_{j} + \tilde{g}_{j - 1} } \right)}}{4} - \varepsilon_{3} Pr\gamma^{*} \left( {\frac{{{\Theta }_{j} + {\Theta }_{j - 1} }}{2}} \right) \\ & \quad + \left( {\tilde{g}_{j - 1} - \tilde{g}_{j} } \right) - \frac{{\varepsilon_{1} }}{{\varepsilon_{4} }}\frac{{PrEcx_{j} (\tilde{v}_{2j} + \tilde{v}_{2j - 1} )^{2} }}{4} - \frac{1}{{\varepsilon_{4} }}\frac{{MPrEcx_{j} (\hat{v}_{1j} + \hat{v}_{1j - 1})^{2} }}{4}. \\ \end{aligned} \right\}$$

The linearized set of equations possess the subsequent block- tridiagonal structure

31 $$A\delta = d,$$

wherein

$$A = \left[ {\begin{array}{*{20}c} {\left[ {A_{1} } \right]} & {\left[ {C_{1} } \right]} & {} & {} & {} & {} & {} \\ {} & {\left[ {A_{2} } \right]} & {\left[ {C_{2} } \right]} & {} & {} & {} & {} \\ {} & {} & {} & \ddots & {} & {} & {} \\ {} & {} & {} & \ddots & {} & {} & {} \\ {} & {} & {} & \ddots & {} & {} & {} \\ {} & {} & {} & {} & {\left[ {B_{J - 1} } \right]} & {\left[ {A_{J - 1} } \right]} & {\left[ {C_{J - 1} } \right]} \\ {} & {} & {} & {} & {} & {\left[ {B_{J} } \right]} & {\left[ {A_{J} } \right]} \\ \end{array} } \right],$$

$$\Delta = \left[ {\begin{array}{*{20}c} {\left[ {{ }\Delta_{1 } } \right]} \\ {\left[ {{ }\Delta_{2 } } \right]} \\ \vdots \\ \vdots \\ \vdots \\ {\left[ {\Delta_{{{\text{J}} - 1 }} } \right]} \\ {\Delta_{{{\text{J}} }} } \\ \end{array} } \right]\;{\text{and}}\;d = \left[ {\begin{array}{*{20}c} {\left[ {\left( {R_{1} } \right)_{{j - \frac{1}{2}}} } \right]} \\ {\left[ {\left( {R_{2} } \right)_{{j - \frac{1}{2}}} } \right]} \\ \vdots \\ \vdots \\ \vdots \\ {\left[ {(R_{{ {\text{J}} - 1}} )_{{j - \frac{1}{2}}} } \right]} \\ {\left[ {(R_{{ {\text{J}}}} )_{{j - \frac{1}{2}}} } \right]} \\ \end{array} } \right].$$

Next, we factorize matrix A into

32 $$A = LU,$$

wherein

$$L = \left[ {\begin{array}{*{20}c} {\left[ {\alpha_{1} } \right]} & {} & {} & {} & {} \\ {\left[ {\Omega_{2} } \right]} & {\left[ {\alpha_{2} } \right]} & {} & {} & {} \\ {} & {} & \ddots & {} & {} \\ {} & {} & \ddots & {\left[ {\alpha_{J - 1} } \right]} & {} \\ {} & {} & {} & {\left[ {\Omega_{J} } \right]} & {\left[ {\alpha_{J} } \right]} \\ \end{array} } \right],\;U = \left[ {\begin{array}{*{20}c} {\left[ I \right]} & {\left[ {\chi_{1} } \right]} & {} & {} & {} \\ {} & {\left[ I \right]} & {\left[ {\chi_{2} } \right]} & {} & {} \\ {} & {} & \ddots & \ddots & {} \\ {} & {} & {} & {\left[ I \right]} & {\left[ {\chi_{J - 1} } \right]} \\ {} & {} & {} & {} & {\left[ I \right]} \\ \end{array} } \right],$$

in which ${\Delta }$ represent the unknowns and $\left[ I \right]$, $\left[ {\alpha_{i} } \right]$, $\left[ {{\Omega }_{i} } \right]$ and $\left[ {\chi_{i} } \right]$ are $6{ \star }6$ matrices. We run the simulations until ${\Upsilon }_{max} = 12$ for the similarity variable. In calculations, a grid-size of $x_{j} = 0.001$ is remarked to be proper and an error-tolerance has been supposed as $10^{ - 6}$. In the current investigation, a consistent mesh of size $x_{j} = 0.001$ is found to assure the convergence and the results are achieved through an error of tolerance $10^{ - 5}$ in all instances. We had assimilated our outcomes with the present literary work of Chen et al.^28^ and Narayana et al.^29^ underneath convinced conditions are met and had observed a remarkable agreement with that literature (see Table 3). This comparison has given us assurance in further results.

**Table 3:** Comparing of ${\Theta }^{{\prime }} \left( 0 \right)$ values when $Ec = M = \beta = \phi = 0$.

$Pr$	Present study	Ref.^28^	Ref.^29^
1	1.3333	1.33334	1.3333
5	2.3801	2.30796	2.3080
10	4.7968	4.79686	4.7969

## Results and discussions

**Figure 4:**
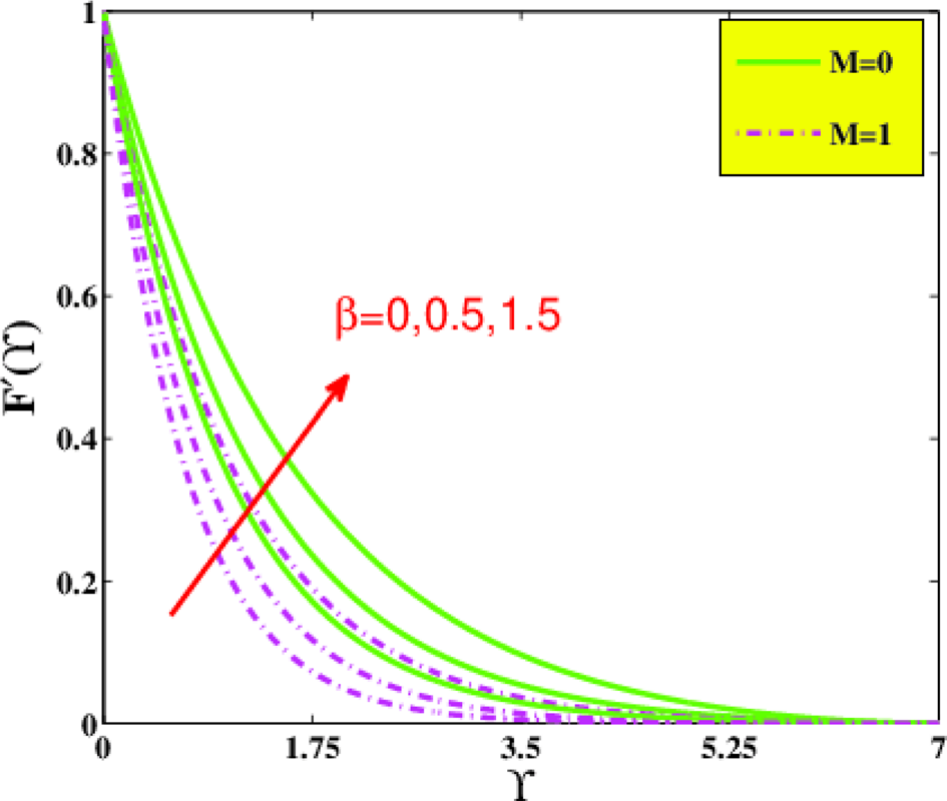
Velocity $F^{\prime}\left( {\Upsilon } \right)$ via $\beta$ and $M$.

**Figure 5:**
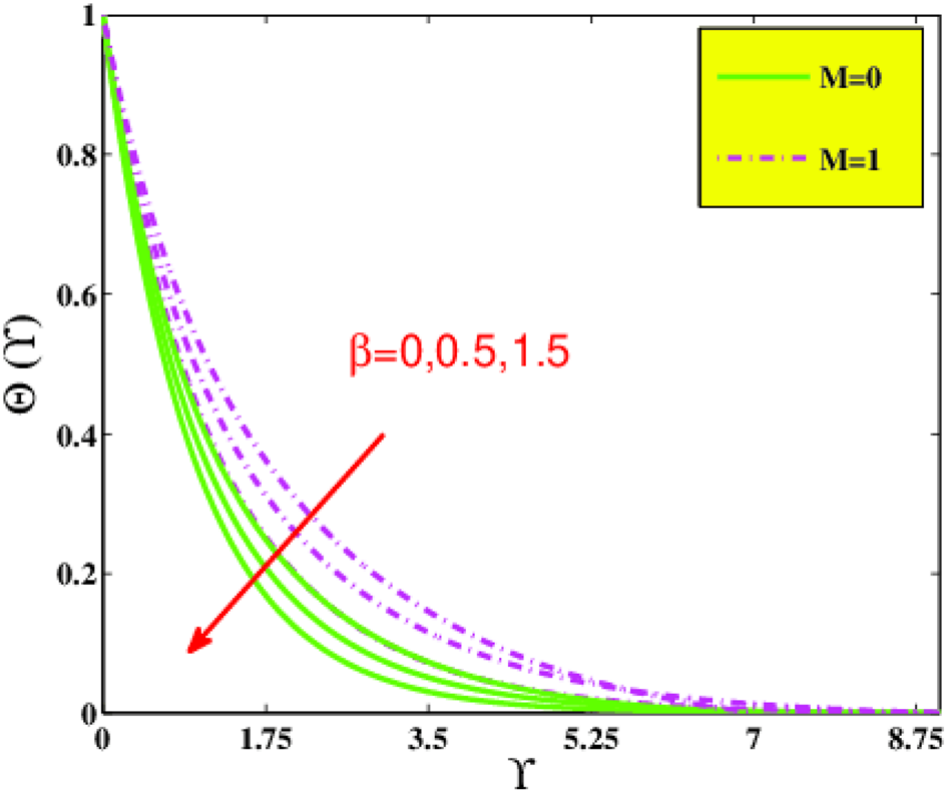
Temperature $\Theta \left( {\Upsilon } \right)$ via $\beta$ and $M$.

**Figure 6:**
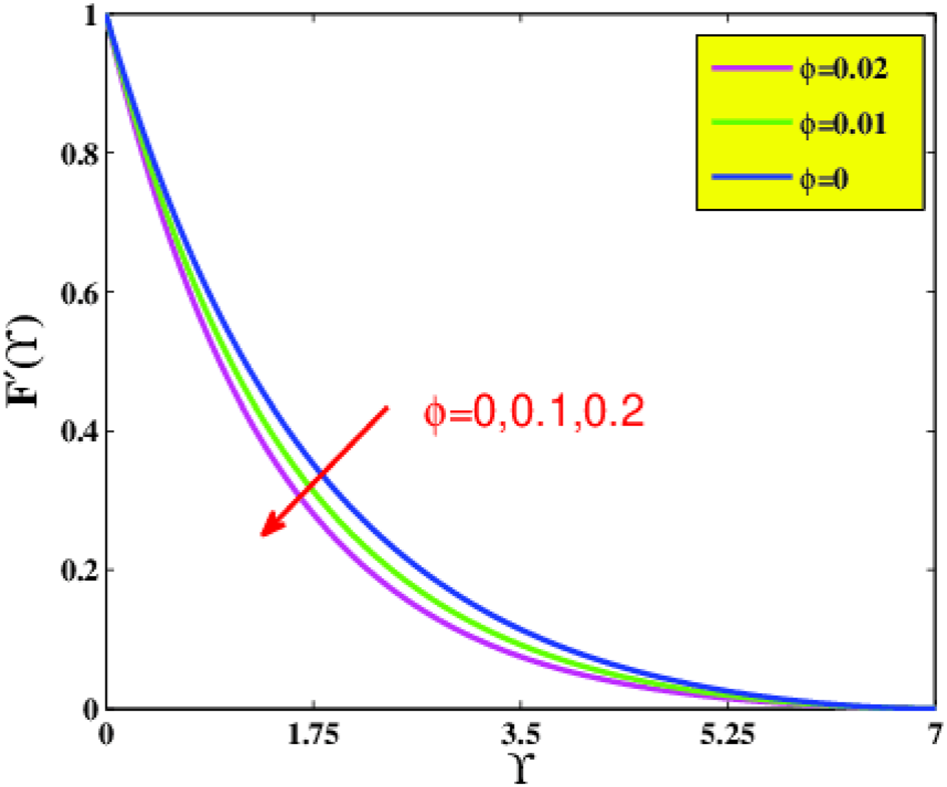
Velocity $$F^{\prime}\left( {\Upsilon } \right)$$ via $$\phi$$.

**Figure 7:**
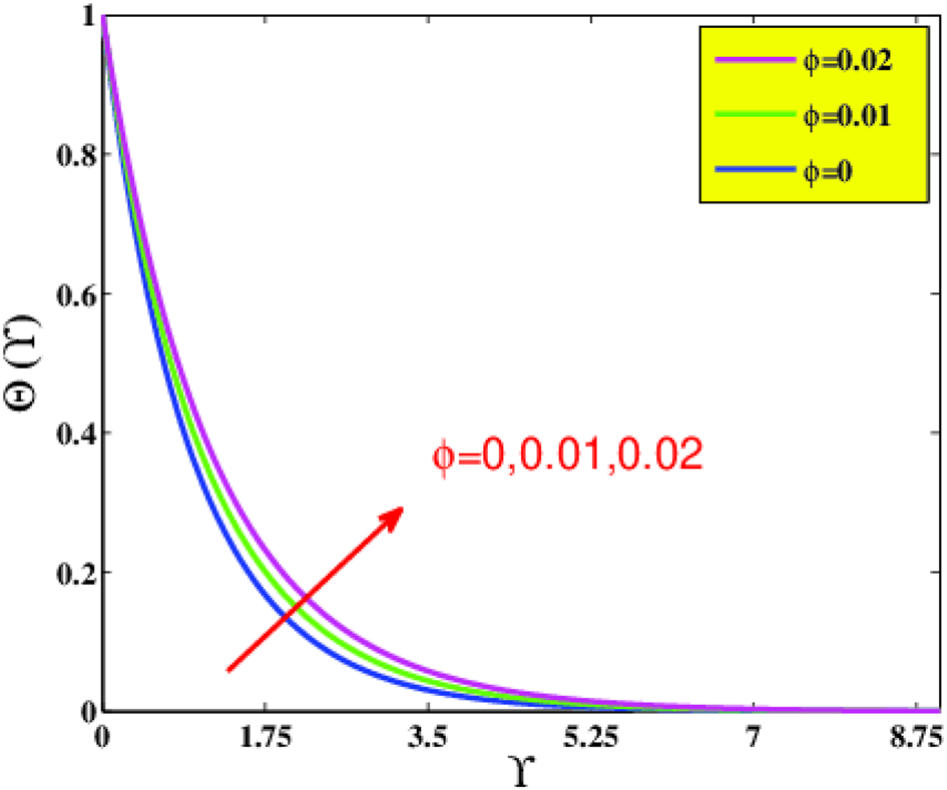
Temperature $$\Theta \left( {\Upsilon } \right)$$ via $$\phi$$.

In the present sector, the physical significance of renowned factors like concentration of nanoparticles $\phi \left( {0.0 \le \phi \le 0.1} \right)$, magnetic field parameter $M\left( {0.0 \le M \le 1} \right)$, Prandtl number $Pr\left( {0.5 \le Pr \le 9} \right)$, Deborah number $\beta \left( {0.0 \le \beta \le 1.5} \right)$, Eckert number $Ec\left( {0.5 \le Ec \le 2.5} \right)$, heat source/sink parameter $\gamma^{*} \left( { - 0.3 \le \gamma^{*} \le 0.3} \right)$ and Jeffrey fluid parameter $\lambda_{1} \left( {0.1 \le \lambda_{1} \le 0.9} \right)$ against velocity, temperature, the drag coefficient, and the heat transference rate is examined through Figs. 4, 5, 6, 7, 9, 10, 11, 12, 13. Table 4 illustrates computational values of drag force $Re_{x}^{1/2} C_{f}$ and heat transference rate $Re_{x}^{ - 1/2} Nu_{x}$ via graphene/EG nanofluid for higher estimation of the concentration of nanomolecules $\phi$, magnetic field $M$, Deborah $\beta$, Eckert $Ec$, and Prandtl $Pr$ numbers. There is a diminution in the magnitude of $Re_{x}^{1/2} C_{f}$ for larger $\phi$ and $M$ although it embellishes for larger $\beta$. It is also noted that for larger estimation of $\phi$ and $\beta$ the heat transference rate enhances while for increasing $Pr$, $Ec$ and $M$ it diminishes.

**Table 4:** Computational amounts of $C_{f}$ and $Nu_{x}$ for different choices of novel factors.

$\phi$	$\beta$	$M$	$Pr$	$Ec$	$Re_{x}^{1/2} C_{f}$	$Re_{x}^{ - 1/2} Nu_{x}$
0.0	0.5	0.5	0.7	0.3	0.2786	2.3967
0.05					0.2157	2.8250
0.1					0.1450	3.2767
0.05	0.0	0.2	0.7	0.3	0.6498	2.7488
	0.5				0.8968	3.0275
	1.0				1.0444	3.1798
0.05	0.2	0.0	0.7	0.3	1.1515	3.4733
		0.5			0.9788	2.9922
		1.0			0.8304	2.5469
0.05	0.5	0.5	0.5	0.3	0.9539	4.5684
			1.0		0.9539	3.7008
			1.5		0.9539	2.6658
0.05	2.0	0.5	0.7	0.5	0.9053	3.0130
				1.0	0.9053	1.7572
				1.5	0.9053	0.9061

Figure 4 delineates for velocity distribution $f^{\prime } \left( {\Upsilon } \right)$ versus the Jeffrey fluid variable $\beta$ and magnetic parameter $M$. It is remarked that by reinforcing the amounts of the Deborah number $\beta$ the magnitude of the momentum inclines to increase. Physically higher Jeffrey parameter implies the material behavior as a non-Newtonian regime, gradually predominated by elasticity and indicating solid-like behavior. It is determined that the creation of wall parallel resistive Lorentz force under the magnetic force field enhances the opposition in the flow field. Figure 4 displays that the axial velocity profile declines with a boost in $M$. It is determined that the creation of wall parallel resistive Lorentz force under the magnetic force field enhances the opposition in the flow field. Figure 5 unfolds the impacts of $\beta$ and $M$ on the temperature field. It has been perceived that a boost in $\beta$ abates the temperature and the thickness of the thermal boundary layer. As $\beta$ has an association with the retardation time, so a rise in the $\beta$ causes the boost in retardation time. Consequently, it reduces the temperature of the fluid. The relation among thermal field ${\Theta }$ and magnetic field $M$ is directly related. The growth in the temperature is because of the impending body force which causes more resistance to the fluid flow. The magnetic parameter has an association with operative Lorentz-force, which produces resisting in the liquid flowing, as a consequence, heat is generated leading to embellishment in $\Theta$.

Based on Fig. 6, on incorporating more nanoparticle size $\phi$ of graphene nano solid-particles in the ethylene glycol standard liquid, the laminar motion profiles of graphene–EG nanofluid diminish for spherical shape nanoparticles. In physical terms, amplification in the strength of $\phi$ leads to advanced concentration of graphene nanomolecules in standard liquid. Consequently, a greater amount of graphene nanoparticles in base fluid boosts resistance to flow, which causes a reduction in a fluid motion. The effect of solid volumetric fraction $\phi$ on temperature field $\Theta \left( {\Upsilon } \right)$ is examined in Fig. 7. As anticipated, the graph supports a substantial increase in temperature by adding more graphene nanoparticles into ethylene glycol. In physical terms, a rise in thermal conductivity was found, which enhances the thermal distribution.

**Figure 8:**
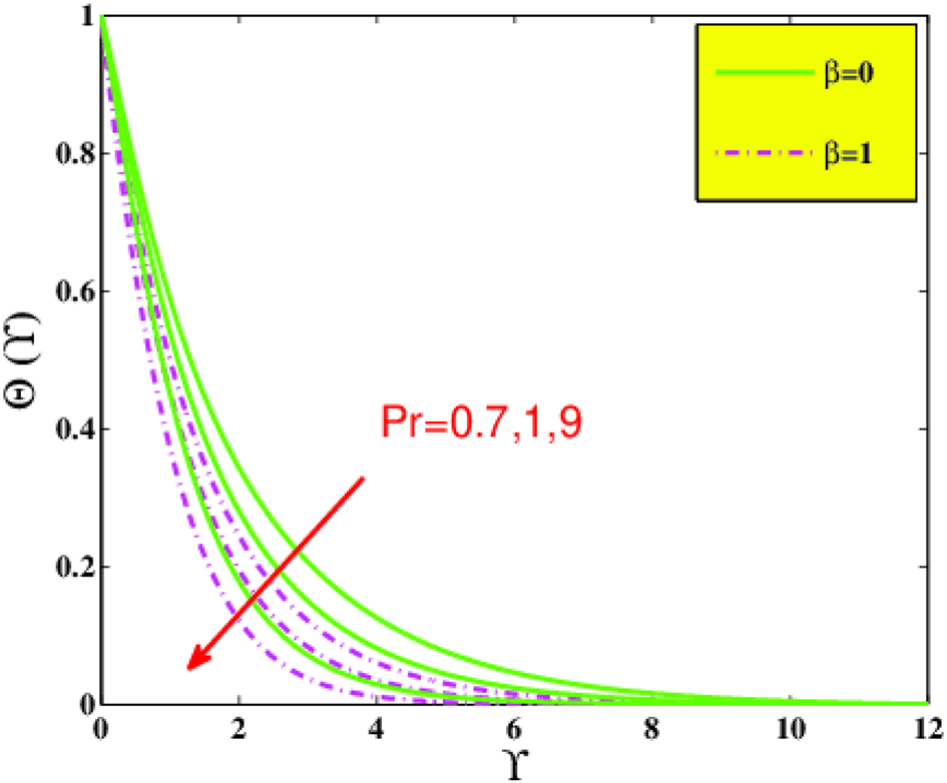
Temperature ${\Theta }\left( {\Upsilon } \right)$ via $Pr$ and $\beta$.

**Figure 9:**
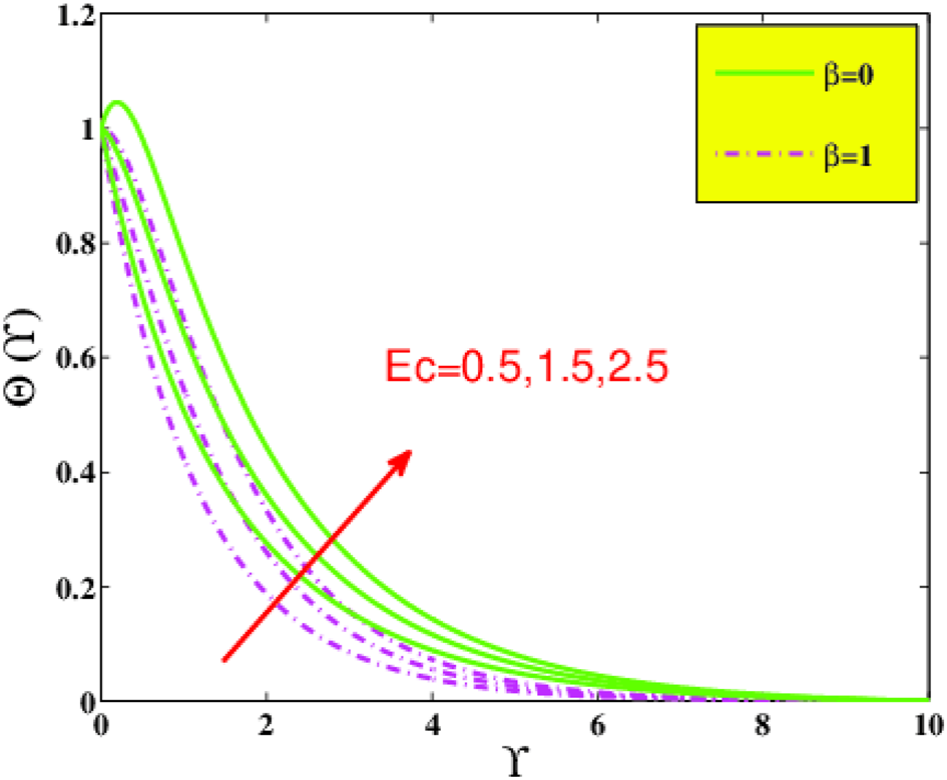
Temperature ${\Theta }\left( {\Upsilon } \right)$ via $Ec$ and $\beta$.

The temperature field ${\Theta }\left( {\Upsilon } \right)$ under the action of Prandtl number $Pr$ is represented in Fig. 8 for various values of $\beta$. As $Pr$ is momentum diffusivity over thermal diffusivity. With an increase in $Pr$ temperature difference reduces this means that thermal diffusivity tends to decrease, as a result, temperature $\Theta \left( {\Upsilon } \right)$ of the nanofluid declines. The variation of viscous heat dissipation also named Eckert number $Ec$ on temperature field $\Theta \left( {\Upsilon } \right)$ is portrayed in Fig. 9. The augmenting of temperature appears with a rise in $Ec$. That is because the friction between fluid layers plays a vital part to magnify the measure of heat in fluid, as heat energy is stored in the nanofluid throughout the entire process.

**Figure 10:**
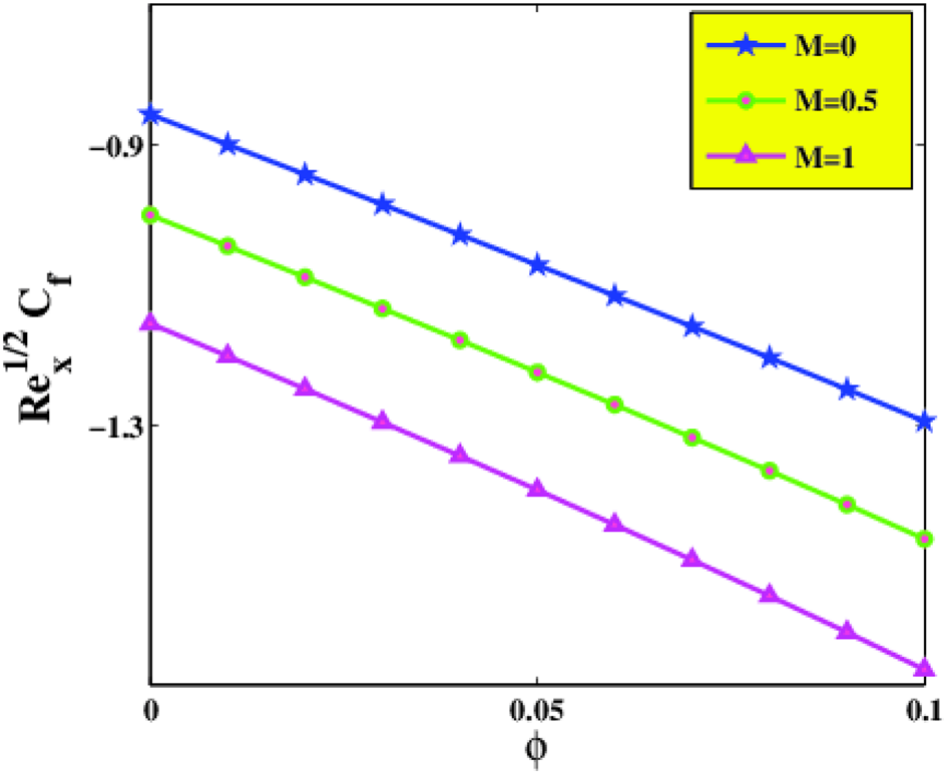
Skin friction $C_{f}$ via $\phi$ and $M$.

**Figure 11:**
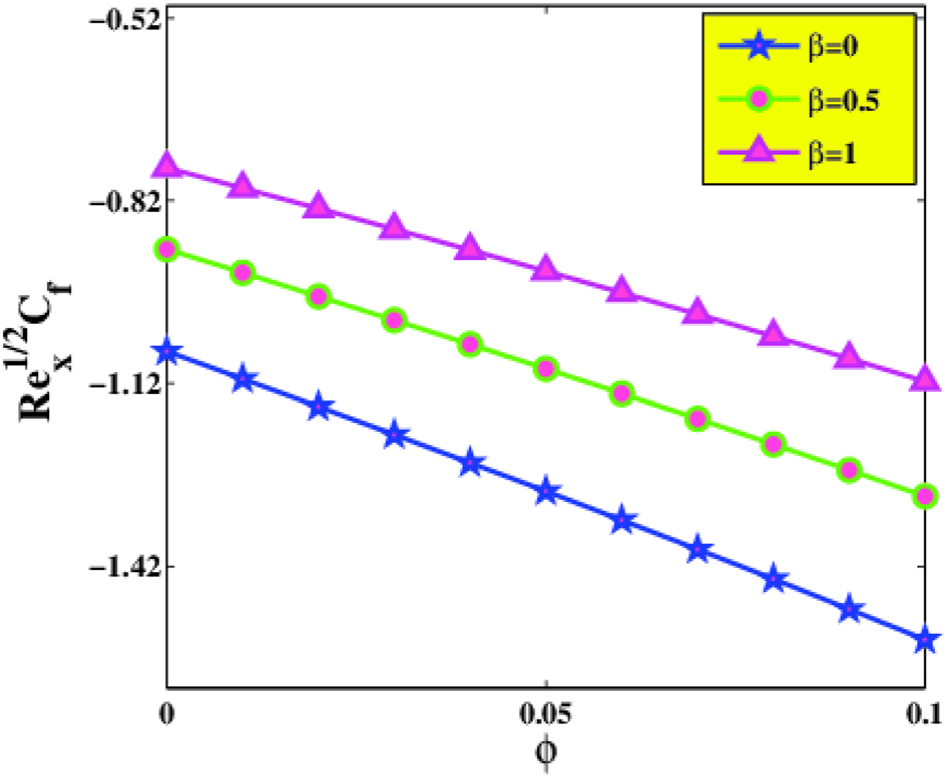
Skin friction $C_{f}$ via $\phi$ and $\beta$.

The variation of skin friction coefficient $Re_{x}^{\frac{1}{2}} C_{f}$ versus $\phi$ and $M$ is illustrated in Fig. 10. As described in the graph, it is remarked that higher values of $\phi$ and $M$ diminish $Re_{x}^{\frac{1}{2}} C_{f}$ at the surface of the sheet. Figure 11 is designed to guess the comportment of Deborah number $\beta$ and concentration of nanoparticles $\phi$ on $Re_{x}^{\frac{1}{2}} C_{f}$. It is discovered that drag force exhibits increasing behavior for incrementing amounts of $\beta$ but declines in the status of $\phi$. As the Deborah number $\beta$ is exploited to elucidate the visco-elastic attribute of a material.

**Figure 12:**
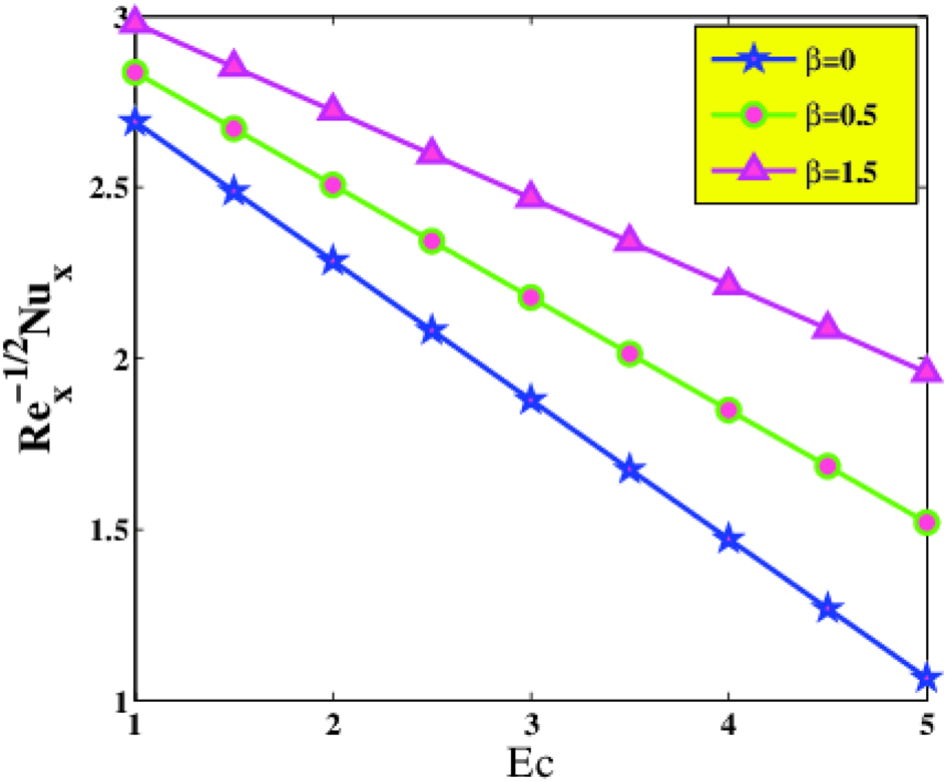
Nusselt number $Nu_{x}$ via $Ec$ and $\beta$.

**Figure 13:**
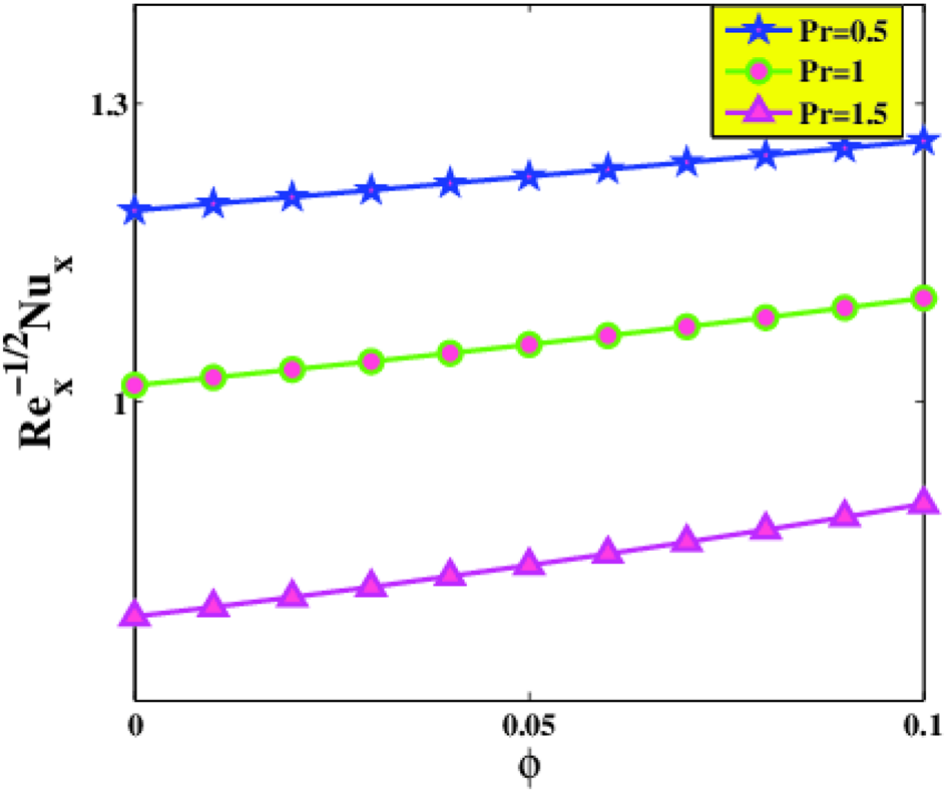
Nusselt number $Nu_{x}$ via $\phi$ and $Pr$.

The change of local Nusselt number $Re_{x}^{{ - \frac{1}{2}}} Nu_{x}$ with various parameters is displayed in Figs. 12 and 13. It is remarked that the heat transference rate diminishes with a boost in Eckert and Prandtl numbers. In the case of a larger $Pr$, nanoliquid thermal diffusivity is declined, accordingly, the heat transport rate lessens. As expected, the local Nusselt number $Re_{x}^{{ - \frac{1}{2}}} Nu_{x}$ increase with increasing Deborah number $\beta$ and nanomolecules size $\phi$. The reason is that the increment of $\beta$ intensifies the fluid elasticity within the flow vicinity. By the accretion of volume fraction of nanoparticles, the collisions of nanoparticles aggravates and assists to augmentation of turbulence intensity of the mixture. The augmentation of turbulence intensity strengthens the advection energy transfer inside the working mixture. In view of this, the energy transfer coefficient increases and leads to enhancement of heat transfer rate.

## Main findings

Numerical scrutiny of MHD graphene-EG nanofluid flow above a stretchable surface with viscidness heat dissipative flow and Ohmic heating influences is explored. The most important consequences of the existing investigation are as described below:

- The graphene-EG Jeffrey nanofluid velocity gets decreased owing to augmentation in nanoparticle size $\phi$ and magnetic force $M$ parameters while the behavior of velocity profile gets overturned due to Jeffrey parameter $\beta$.
- Prandtl number $Pr$ and Jeffrey parameter $\beta$ parameters diminish the temperature of Jeffrey nanoliquid in the boundary-layer regime whereas the fluid temperature is enhanced owing to a rise in concentration, Lorentz force, and viscous heat dissipation parameters.
- The Local Nusselt Number of graphene-EG Jeffrey nanofluid is reduced owing to $\beta$, $Pr$, and $Ec$ parameters though it is augmented result in an upsurge in nanoparticles volume fraction parameter.
- The graph emphasizes that both conventional fluid and nanofluid are good in heat transsport rate. By comparing, graphene-EG nanofluid yield high heat transport rate in comparison with base fluid.
- Lorentz force and concentration diminish the frictional force at the stretchable plate.
- The acquired outcomes depict that both base fluid and nanofluid offer positive response on shear stress. By comparing, convectional fluid holds better augmentation of shear stress in comparison with to graphene-EG nanofluid.

## List of symbols

$$v_{1} ,v_{2}$$: Velocity components
$${{\yen}}$$: Temperature field
$$C_{p}$$: Specific heat
$C_{f}$: Skin friction coefficient
$${{\yen}}_{w}$$: Wall temperature
$${{\yen}}_{\infty }$$: Ambient temperature
$f^{\prime }$: Non-dimensional velocity
$$L$$: Characteristic length
$$v_{1w}$$: Wall velocity
$M$: Magnetic field parameter
*Nu*: Nusselt number
*Pr*: Prandtl number
*Ec*: Eckert number
*a*: Extending rate
$q_{w}$: Wall heat flux
$$k$$: Thermal conductivity
$B_{0}$: Magnetic field strength

## Greek symbols

$$\nu$$: Kinematic viscosity
$\rho$: Density of fluid
$$\beta$$: Deborah number
$${\Upsilon }$$: Similarity $$z$$ variable
$$\Theta$$: Dimensionless temperature
$$\psi$$: Stream function
$$\alpha$$: Thermal diffusivity
$$\sigma$$: Electric conductivity
$$\mu$$: Dynamic viscosity
$$\lambda_{1}$$: Relaxation time
$$\lambda_{2}$$: Retardation time
$\tau_{w}$: Wall shear-stress
$$\phi$$: Size concentration

## Subscripts

$$nf$$: Nanofluid
$$f$$: Fluid phase
$$s$$: Solid phase
$$p$$: Nanoparticles
